# Comment on “A global environmental crisis 42,000 years ago”

**DOI:** 10.1126/science.abi8330

**Published:** 2021-11-18

**Authors:** Andrea Picin, Stefano Benazzi, Ruth Blasco, Mateja Hajdinjak, Kristofer M. Helgen, Jean-Jacques Hublin, Jordi Rosell, Pontus Skoglund, Chris Stringer, Sahra Talamo

**Affiliations:** 1 Department of Human Evolution, Max Planck Institute for Evolutionary Anthropology, Deutscher Platz 6, Leipzig 04103, Germany; 2 Department of Cultural Heritage, University of Bologna, Via Degli Ariani 1, Ravenna 48121, Italy; 3 Institut Català de Paleoecologia Humana i Evolució Social (IPHES-CERCA), Zona Educacional 4, Campus Sescelades URV (Edifici W3), Tarragona 43007, Spain; 4 Departament d’Història i Història de l’Art, Universitat Rovira i Virgili, Avinguda de Catalunya 35, Tarragona 43002, Spain; 5 Department of Evolutionary Genetics, Max Planck Institute for Evolutionary Anthropology, Deutscher Platz 6, Leipzig 04103, Germany; 6 Francis Crick Institute, 1 Midland Rd, London NW1 1AT, UK; 7 Australian Museum, 1 William Street, Sydney NSW 2010, Australia; 8 Chaire de Paléoanthropologie, Collège de France, 11 Place Marcelin Berthelot, Paris 75231, France; 9 CHER, Department of Earth Sciences, Natural History Museum, London SW7 5BD, UK; 10 Department of Chemistry “G. Ciamician”, University of Bologna, Via Selmi 2, Bologna 40126, Italy

## Abstract

Cooper *et al*. (Research Articles, 19 February 2021, p. 811) propose that the Laschamps geomagnetic inversion ~42 ka BP drove global climatic shifts, causing major behavioural changes within prehistoric groups, and events of human and megafaunal extinction. Other scientific studies indicate that this proposition is unproven from the current archaeological, paleoanthropological, and genetic records.

Cooper and colleagues recently reported a tree-ring based ^14^C dataset (42 to 36 ka ^14^C BP) based on four Kauri trees, achieving high precision data (±107 to 180 yr, 1-σ), ideal to reconstruct the increase of ^14^C production during the Laschamps excursion, and create a detailed calibration curve Kauri-Hulu (1). These data allowed the authors to model statistically possible variations of the global climate during the geomagnetic inversion. Although we appreciate the scientific advances accomplished in (1), we note with concern several statements relating the supposed impacts of the Laschamps on hominin and faunal extinctions and human behavioural changes, which misconstrue the current paleontological, archaeological, and genetic data. Geomagnetic reversals were frequent during the Pliocene and Pleistocene (2), and mass extinctions at the time of these inversions have not been documented in the paleontological and archaeological record so far. For example, the Blake excursion (~114 ± 1 ka BP) (3) occurred without apparent serious effects on the subsistence of Neanderthals in Eurasia, *Homo sapiens* in Africa, and megafauna in Australia. In our view, Cooper and colleagues have used the archaeological and paleontological data selectively in order to create a narrative that could support the Laschamps as the main driver of a global environmental crisis. Here, we contextualize the evidence at ~45-40 ka BP to show that the claimed huge impacts of the geomagnetic inversion on humans and megafauna go far beyond the available data. We observe three main issues in (1) that include the extinction of megafauna in Australia, the demise of Neanderthals and early groups of *Homo sapiens* in Europe, and the emergence of figurative art in caves.

In our view, the Greenland ice cores and marine records do not document any notable effects of the Laschamps excursion on the global climate (4). However, (1) argues that Laschamps-associated changes in climate can be linked to megafaunal extinctions, especially in Australia, which they suggest peaked at 42.1 ka. Recent research now suggests that much of Australia’s megafauna survived beyond 40.1 ka BP (5). While ancestry replacements frequently occurred during the last glacial period in Eurasian megafauna, synchronous bottlenecks or extinctions around 45-40 ka BP have not been noted (6). Although with turnovers, most of these taxa survived the Last Glacial Maximum (e.g. *Coelodonta antiquitatis*) and even the Pleistocene-Holocene transition (e.g. *Mammuthus primigenius*).

The second main issue of (1) is the presumed relation between the climatic impact of the Laschamps and the extinction of Neanderthals and contemporaneous European *Homo sapiens*. We clarify that during their evolutionary history, Neanderthals survived glaciation events and climatic fluctuations harsher than the stadials GS-11 and GS-10 (7). During Marine Isotope Stage (MIS) 6 and MIS 4, the Scandinavian ice sheet reached Central Germany and the coast of Poland, respectively. Therefore, climate change may only have played a minor role in the fate of the Neanderthals (8). A more likely factor is gradual competitive exclusion, caused by the dispersals of *Homo sapiens* in Europe after ~46 ka BP (9), which disrupted the Neanderthal niche structure and food web.

Additionally, the radiocarbon dataset used by (1) (see Fig. S31) for establishing the temporal range of Neanderthals’ demise is arbitrary in the selection of ^14^C dates. A better solution would have been to compare the chronological boundaries of key sites or the direct dates of human fossils (Fig. 1 and Table 1). In Iberia, Neanderthals may have persisted after a threshold of ~40 ka BP ((10) and references therein), while the chronology of the last Neanderthals in Central and West Asia is still virtually unknown. Moreover, we note that the end of the Middle Palaeolithic at one or a group of sites does not necessarily reflect the end of Neanderthals as a species, and current scenarios may change with further research in less investigated areas.

The claim that the Laschamps event had a negative impact on some early European *Homo sapiens* populations is also problematic. If the weakened geomagnetic field allowed a rise in ultraviolet radiation in equatorial and low latitudes, *Homo sapiens* in Africa should have been even more affected than groups living in temperate environments. Hence, the Laschamps should have slowed the dispersal out of Africa and beyond, whereas data suggest that it had no such effect. Similarly, no large-scale impact at ~42 ka BP is observed in the known African archaeological, paleoanthropological, or genetic records (11).

Furthermore, if we consider both the short (Uluzzian: 45/43-40 ka cal BP; Protoaurignacian: 41.5-39.9 ka cal BP; Early Aurignacian: 39.8-37.9 ka cal BP) and the long (Early Aurignacian: 42.5-37.9 ka cal BP) chronology for the cultural succession of the Early Upper Palaeolithic (12), we note that *Homo sapiens* certainly survived the climatic consequences of the Laschamps. This evidence makes it unclear how ultraviolet radiation affected only some European inhabitants when no data currently support the greater use of ochre as sunscreen in the Aurignacian or any other Upper Palaeolithic culture. In addition, although the end of the Uluzzian temporally overlapped with the Protoaurignacian in northern Italy (13), the lamellar technologies of the Aurignacian may have originated in western Asia rather than developing from previous technical behaviours of *Homo sapiens* in Europe (12).

Lastly, in the archaeological record, a large increase in the use of caves at 42-40 ka BP is not apparent in the data. Since the Lower Palaeolithic, the occupations of these natural shelters were the results of complex settlement dynamics and subsistence strategies (14). Figurative cave paintings may have emerged as an artistic expression that tried to imitate and transfer natural patterns in new contexts. These behaviours had appeared in eastern Borneo by 52-40 ka BP, in Sulawesi by at least 45.5 ka BP, and possibly in Europe before 64 ka BP ((15) and references therein), a time period well before the increase in the ultraviolet radiation caused by the Laschamps event.

All in all, not only have Cooper and colleagues failed to provide convincing explanatory mechanisms relating the Laschamps excursion to cultural and biological changes, but their chronological coincidence with this geomagnetic reversal is highly questionable.

## Figures and Tables

**Figure 1:**
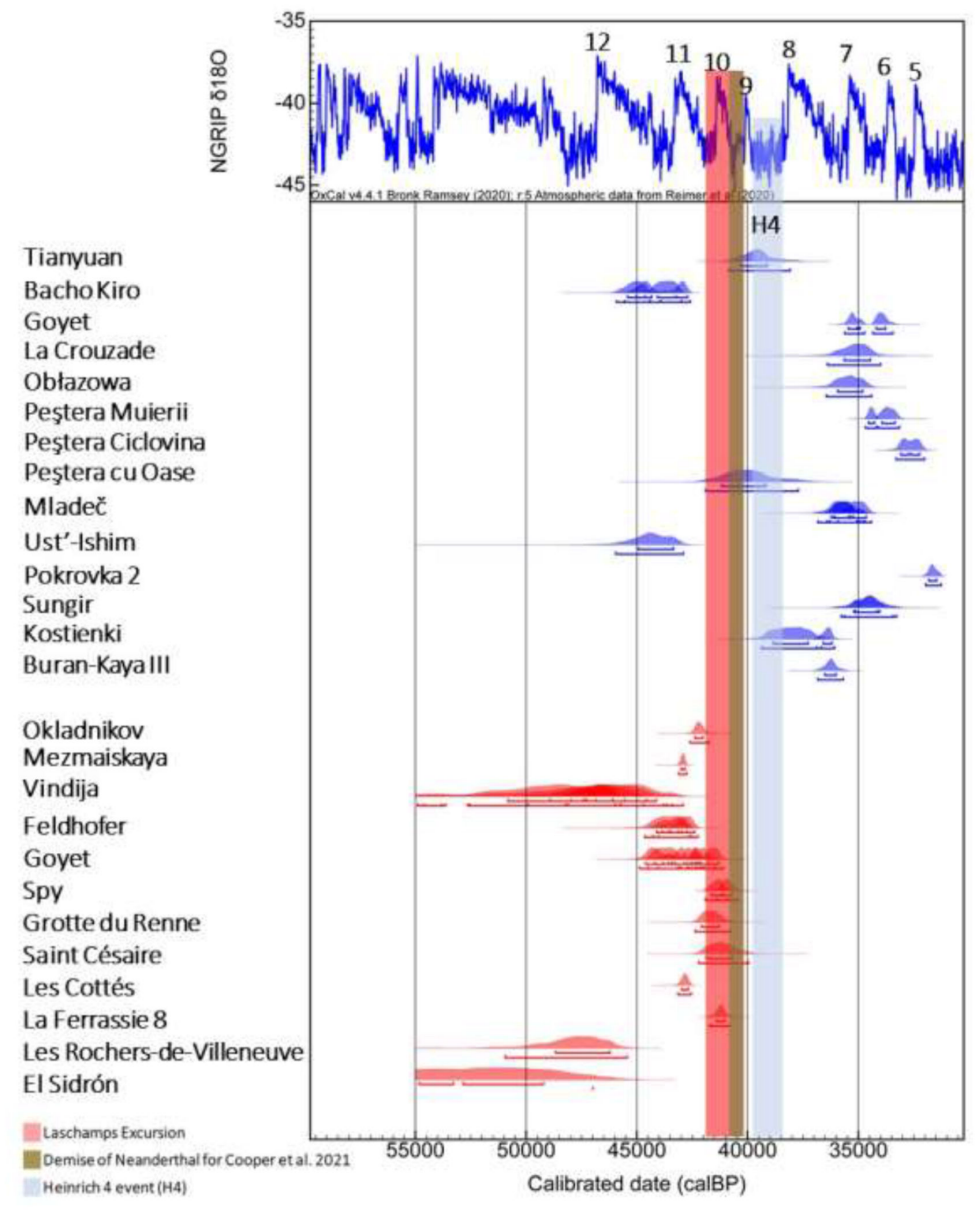
Neanderthals and *Homo sapiens*' direct date*s* published before the Cooper et al. 2021 paper. Some hominins have more than one date (Spy, Goyet, Kleine Feldhofer, Vindija, Kostienki, Sungir, Peştera Mureii, Mladeč and Bacho Kiro), and are merged together in one single line in the graph. The calibrated ranges are produced using IntCal 20 in the OxCal 4.4 program (P. J. Reimer *et al*., The INTCAL20 northern hemisphere radiocarbon age calibration curve (0–55 cal kBP). *Radiocarbon*, 1-33 (2020); C. B. Ramsey, Bayesian analysis of radiocarbon dates. *Radiocarbon* **51**, 337-360 (2009)).

## Data Availability

Table 1 can be downloaded from Zenodo (XXX).
